# Two DNA Aptamers against Avian Influenza H9N2 Virus Prevent Viral Infection in Cells

**DOI:** 10.1371/journal.pone.0123060

**Published:** 2015-03-31

**Authors:** Yuewei Zhang, Ziqiang Yu, Fei Jiang, Ping Fu, Junjun Shen, Wenxue Wu, Jinxiang Li

**Affiliations:** 1 Laboratory of Rapid Diagnostic Technology for Animal Disease, College of Veterinary Medicine, China Agricultural University, Beijing, P. R. China; 2 Key Laboratory of Animal Epidemiology and Zoonosis, Ministry of Agriculture, College of Veterinary Medicine, China Agricultural University, Beijing, P. R. China; 3 Chinese Academy of Agricultural Sciences, Beijing, P. R. China

## Abstract

New antiviral therapy for pandemic influenza mediated by the H9N2 avian influenza virus (AIV) is increasingly in demand not only for the poultry industry but also for public health. Aptamers are confirmed to be promising candidates for treatment and prevention of influenza viral infections. Thus, we studied two DNA aptamers, A9 and B4, selected by capillary electrophoresis-based systemic evolution of ligands by exponential enrichment (CE-SELEX) procedure using H9N2 AIV purified haemagglutinin (HA) as target. Both aptamers had whole-virus binding affinity. Also, an enzyme-linked aptamer assay (ELAA) confirmed binding affinity and specificity against other AIV subtypes. Finally, we studied aptamer-inhibitory effects on H9N2 AIV infection in Madin–Darby canine kidney (MDCK) cells and quantified viral load in supernatant and in cell with quantitative PCR (qPCR). Our data provide a foundation for future development of innovative anti-influenza drugs.

## Introduction

Aptamers are single-stranded oligonucleotides isolated from *in vitro* selection process called systematic evolution of ligands by exponential enrichment (SELEX) [1–2]. After iterative cycles of library-target incubation, affinity separation of candidate oligonucleotides and PCR amplification, candidates with high binding affinity are identified from an initial oligonucleotide pool containing a region of randomized 30–100 nucleotides. Aptamers, which can fold into different three-dimensional structures, can bind to diverse targets with high affinity and specificity [3–4], similar to antibody-antigen interactions. However, compared to antibodies, aptamers are more stable, inexpensive, and more easily modified and reproduced, suggesting their suitability for diagnoses and treatment of pathogenic infections [4].

Avian influenza virus (AIV) (*Orthomyxoviridae*, influenza A virus) [5] is comprised of subtypes based on surface glycoproteins, haemagglutinin (HA), and neuraminidase (NA). HA is critical to viral entry, mediating viral attachment to host cells and subsequent membrane fusion [6–7]. Also, HA can induce the neutralizing antibodies production, which can be used to inhibit early phase viral infection [8]. Currently, 17 HA subtypes (H1-H17) of influenza A virus have been identified [9–10]. Since the 1990s, subtype H9 viral infections have been reported in many countries [11–13]. And this subtype is known to infect chickens, ducks, and pigs and other animals as well as humans [14–15]. Thus H9-specific probes such as aptamers may be promising tools for viral diagnosis and treatment.

In addition to drugs [16], antisense oligonucleotides (ASOs) [17], monoclonal antibodies [18], and aptamers are alternative options for prophylaxis and treatment of influenza viral infections. Several recombinant HA proteins or peptides have been used to select aptamers, including recombinant HA proteins of H5 [19–20], peptides of the H9 receptor binding region [21] and peptides of the HA conserved region [22]. In addition, whole influenza virus (H3N2) has been used for aptamer selection and the final aptamers were confirmed to have HA-binding affinity [23]. Furthermore, purified HA proteins (from human influenza B virus) have also been reported to select HA-specific aptamers [24–25].

In the present study, capillary electrophoresis (CE) was used to separate target-bound and unbound DNA sequences. Compared with other separation techniques of conventional SELEX, such as affinity columns and filtering techniques, CE selection is less time consuming, [26–27], allows aptamer-target binding in free solution [28–29], and offers greater separation efficiency [30]. Finally, A9 and B4 aptamers were confirmed to bind the whole virus and efficiently inhibit viral infection in host cells.

## Materials and Methods

### Ethics statement

All animal research was approved by the Beijing Association for Science and Technology (approval ID: SYXK, Beijing, 2007–0025) and by the animal welfare committee of China Agricultural University. All animal studies were in compliance with the Beijing Laboratory Animal Welfare and Ethics guidelines as issued by the Beijing Administration Committee of Laboratory Animals, and were compliant with the China Agricultural University Institutional Animal Care and Use Committee guidelines (ID: SKLAB-B-2010-003).

### Virus and viral proteins

Different subtypes (i.e., H1, H5, H7, and H9) of AIV were used (Table 1). Live viruses were grown in embryonated chicken eggs and subtypes were identified using the corresponding positive serum (WeiKe Biotechnology, Harbin, China). After purification via density-gradient centrifugation, viruses were used to prepare HA as described previously [31]. Purified HA was quantified with a BCA protein assay kit (CWBiotech, Beijing, China) according to the manufacturer’s instructions. In addition, some recombinant HA proteins were also purchased from eENZYME, LLC., USA (Table 1). Both the virus and protein were stored at -80°C until experiments.

**Table 1:** Different AIV subtypes in the Specificity test.

Subtype	Definition	
H5^a^	A/Anhui/a/2005	Recombinant protein
H5^a^	A/goose/Guiyang/337/2006	Recombinant protein
H7^a^	A/Chicken/Netherlands/1/03	Recombinant protein
H7^a^	A/England/268/96	Recombinant protein
H1N1^b^	A/Beijing/501/2009	Virus
H5N1^b^	A/chicken/Henan/1/04	Inactivated Virus
H7N9^b^	A/Anhui/1/2013	Virus
H9N2^b^	A/Chicken/ Beijing/ 1/ 2001	Virus
H9N2^c^	A/Chicken/Hebei/3/98	Virus

a, Purchased from eENZYME, LLC. USA

b, Provided by Associate Professor Jianyu Chang, China Agricultural University.

c, Obtained from Harbin Veterinary Research Institute (HVRI)

### Aptamer selection in CE-SELEX

All capillary electrophoretic experiments were performed using P/ACE MDQ CE equipment (Beckman Coulter, Inc, USA). Bare-fused silica capillaries (75 μm id, 360 μm od, total length of 60.2 cm) were used. Aptamer selection was performed with a previously published CE-SELEX procedure [32]. All oligonucleotides used were synthesized and sequenced at Sangon Biological Engineering Technology and Services.

### Determination of CE separation collection window

Prior to CE-SELEX, the collection window was determined. The ssDNA library in separation buffer (50 mM Tris-HCl, pH 8.3) was heated to 95°C for 10 min, and then cooled to 25°C. The library (0.635 mM) was incubated with 20 μM purified H9 HA (10 μL volume) for 30 min at 25°C. The sample mixture was injected and separated in one CE run using the separation buffer as running buffer. The aptamer-collection window started from the left peak boundary of the ssDNA-HA complex and ended prior to the unbound DNA library peak.

### Binding measurements

Dissociation constant (K_d_) values of all candidate aptamers were confirmed with a previously developed CE-based method [32]. Both purified HA and whole viruses were applied as targets, and aptamers with high binding affinity was confirmed via ELAA with the following procedure. H9-AIV-coated 96-well microplates were blocked with 2% BSA. Then, 100 μl denatured biotin-labeled aptamers (200–0 nM) was added to each well and incubated for 60 min at 25°C. Microplates were washed three times with 50 mM Tris-HCl (pH 8.3) and then incubated with 1:4,000 HRP-SA for 30 min at 25°C. After three washes with 50 mM Tris-HCl (pH 8.3), the substrate TMB (Sigma) was added and incubated for 15 min at 25°C. Then 50 μl sulfuric acid (2 M) was added to stop the reaction. Optical densities were read (450 nm) and the equation Y = B_max_X/(K_d_ + X) was used to calculate K_d_ using GraphPad Prism 5.0 and a saturation curve was obtained. Here, Y represented the mean OD_450 nm_ value, B_max_ was the maximal OD_450 nm_ value, and X was the concentration of the biotin-labeled aptamer. MFOLD [33] was applied to predict secondary structures of the final aptamers binding to H9 AIVs.

### Binding specificity

Proteins and viruses (Table 1) were used to confirm specificity of selected aptamers via ELAA. First, 100 μl of HA (1.32 μM) or viruses with 2^8^ hemagglutination titers were coated, and after blocked with 2% BSA, 100 nM biotin-labeled A9 (or B4) was added and incubated at 25°C. Finally, OD_450_ of each well was read and used to determine the binding specificity of A9 (or B4).

In addition, a competitive ELAA (c-ELAA) confirmed binding specificity. With c-ELAA, only H9N2 AIV was coated and unlabeled aptamers (200–0 nM) with 100 nM biotin-labeled aptamers were simultaneously added into 96-well microplates. Because of the high binding specificity, OD_450_ decreased as unlabeled aptamer concentrations increased.

### Florescent imaging of aptamer-virus binding

First, 20 μl of 0.25% (v/v) red blood cell (RBC) suspension was dropped on the glass slides and absorbed to the surface after incubation at 37°C for 30 min. Then, purified virus solutions (> 2^9^ hemagglutination titers) were added to cover RBCs, followed by 60 min of incubation at 37°C. Next, three FAM labeled ssDNA, aptamer A9, B4 and the control ssDNA (5’-AGT CCG TGG TAG GGC AGG TTG GGG TGA CT-3’), were prepared (100 nM) and incubated with RBC blots at 37°C for 60 min. After each step mentioned three washes with PBS was performed. Finally, cell imaging was performed with a fluorescent microscope (Leica EL 6000) under 488 nm exciting light and visible light.

### Inhibition of the viral infection in MDCK cells

A 2.5-ml aliquot of Madin–Darby canine kidney (MDCK) cells (4 ×10^5^/ml), cultured in Dulbecco’s modified eagle media (DMEM; HyClone Laboratories) containing 10% fetal bovine serum (FBS), were seeded into each well of a six-well plate 24 h before the experiment. Before being added to the MDCK monolayer, four 50% tissue culture infective doses (TCID_50_) of H9N2 viruses per milliliter were incubated with 1 μM A9, B4 and control ssDNA (5’-AGT CCG TGG TAG GGC AGG TTG GGG TGA CT-3’), respectively, in PBS at 25°C for 30 min. Complementary sequences (1 μM) were used to hybridize the corresponding aptamers before viral incubation, and H9N2 viral infection inhibition was compared. After three washes with PBS, the MDCK monolayer was incubated with aptamer-treated viral solutions at 37°C. After one hour of incubation, supernatant was discarded, and cells were washed once with PBS to remove the viruses which did not adhere to RBCs and cultured in ssDNA-free DMEM containing 1% FBS at 37°C.

Viruses were quantified in supernatant and in cells and these were used to calculate aptamer inhibitory activity in different experimental groups. For supernatant samples, viral concentrations were measured. 0.3 ml aliquots of the supernatant were collected at 12, 24, 36, and 48 h, and viral RNA was extracted. For cellular viruses, the ratio of viral load to cell number was measured. Cells were harvested at 4, 8, and 12 h, and cells were counted and total RNA was extracted. Trizol (Invitrogen) was used for RNA extraction according to the manufacturer’s instructions. A quantitative PCR (qPCR) kit, TransScript Green One-Step qRT-PCR SuperMix (TransGen Biotech, Beijing, China), was used to qualify viral RNA.

The primers used in qPCR were 5’-GAA TCC AGA TCT TTC CAG AC-3’ and 5’-CCA TAC CAT GGG GCA ATT AG-3’, as recommended by the World Health Organization (WHO) for detecting subtype H9 AIV. The reaction was performed on an ABI 7500 as follows: 45°C reverse transcription for 10 min, 95°C for 10 min, 40 cycles of denaturation at 95°C for 30 s, annealing at 48°C for 30 s, and extension at 72°C for 40 s. Data analysis was performed using 7500 software, version 2.0 (Applied Biosystems). A recombined plasmid containing the *H9-HA* gene, prepared previously [34], was used as a standard template in this study.

## Results

### Collection window

**Fig 1:**
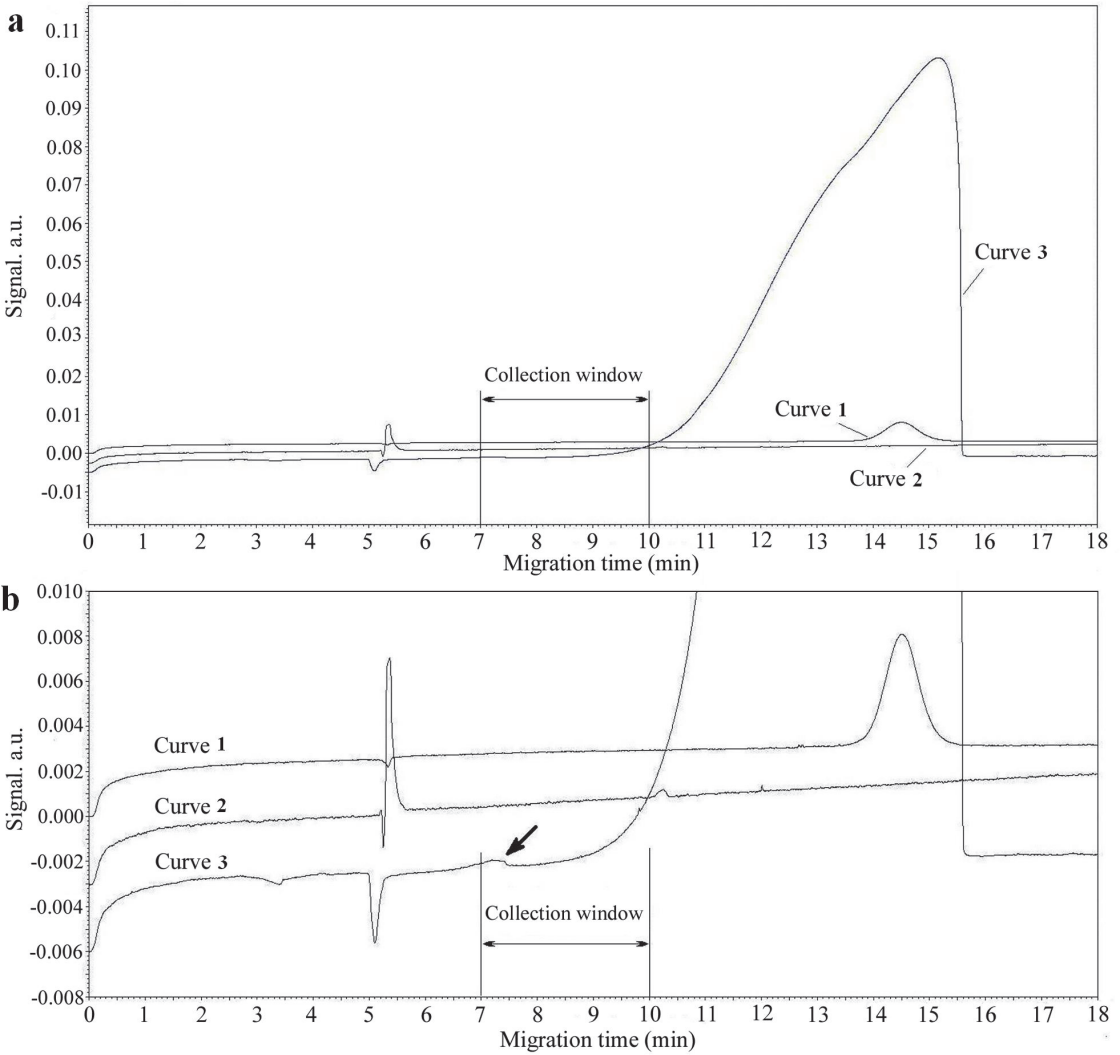
Determination of the aptamer collection window. (a) Whole graph of capillary electrophoregrams for determining the collection window. Curve 1, 2.5 μM library DNA; curve 2, 20 μM purified H9-HA; curve 3, 0.635 mM library DNA with 20 μM protein. (b) Details of (a). The arrow indicates the peak of the complexes, and a collection window from 7 min to 10 min was determined. CE conditions: running buffer, 50 mM Tris-HCl at pH 8.3; injection, 0.5 psi (3,447.5 Pa) for 5s; separation voltage +20 kV. The purified protein solution was detected by UV_280 nm_ and the solutions with aptamers were detected by UV_254 nm_.

We first measured migration time of purified HA protein and free aptamers (Fig 1). Peaks of HA and free aptamers appeared at 5.3 min and 14.5 min, respectively. High concentrations of both protein (0.635 mM) and the library (20 μM) were used to measure complex migration time. A high ssDNA library concentration produced a broad misshapen peak but separation of complex and unbound library was successful. A small complex peak was observed at 7.3 min (Fig 1, curve 3). Thus, the collection window was confirmed to start at 7 min and end at 10 min, and this was used in subsepuent selection rounds.

### Aptamers selected against HA of H9N2 AIV

**Fig 2:**
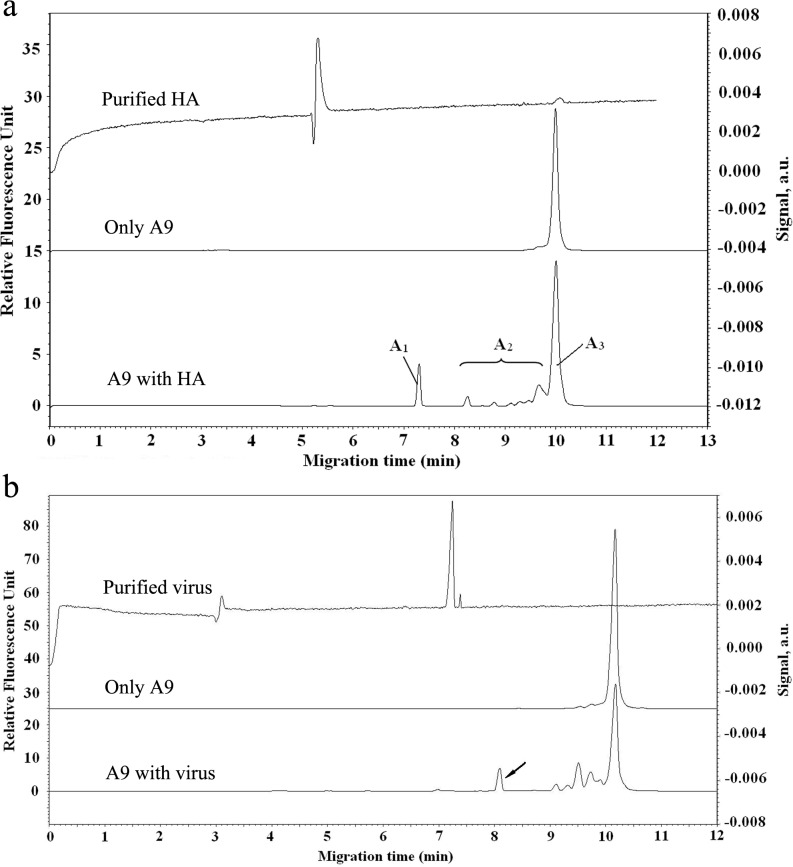
Binding measurements in CE. (a) Electropherogram of A9 binding to the purified H9-HA. The purified HA was detected by a UV detector (280 nm) and had a migration time of 5.4 min. A_1_, A_2_, and A_3_ represent the areas of peaks of the complexes (A_1_), the aptamer dissociated from the complex during electrophoretic separation (A_2_) and the free aptamer (A_3_) respectively. (b) CE detection of the aptamer-virus interaction. The purified H9N2 AIV was detected by a UV detector (280 nm) and had a peak at the migration time of 7.4 min. The arrows showed the aptamer-virus complex at the migration time of 9.1 min. LIF detector was used for all FAM labeled aptamers. K_d_ values were determined with a previously developed CE-based method (see reference [32]).

**Fig 3:**
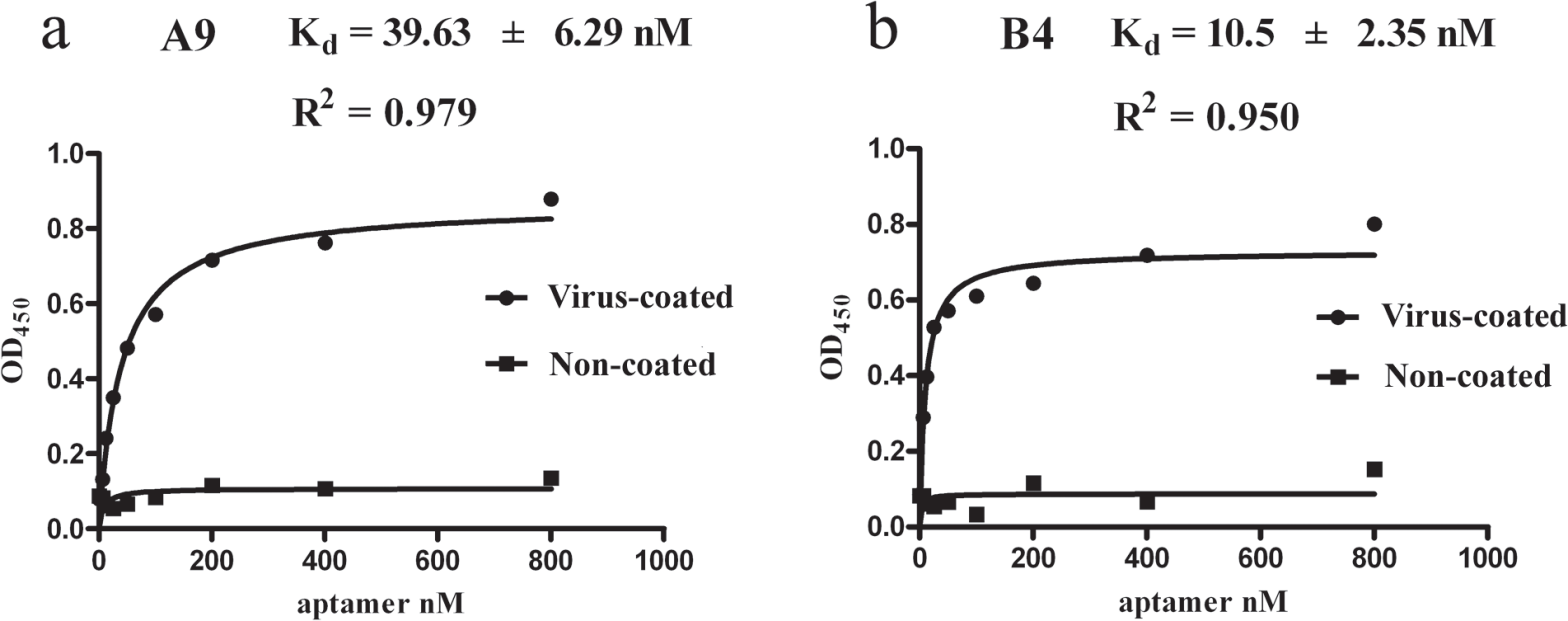
Measurements of binding capability by ELAA. The fitted curves were drawn with the GraphPad Prism 5.0 using various concentrations of aptamers as the binding ligands.

**Fig 4:**
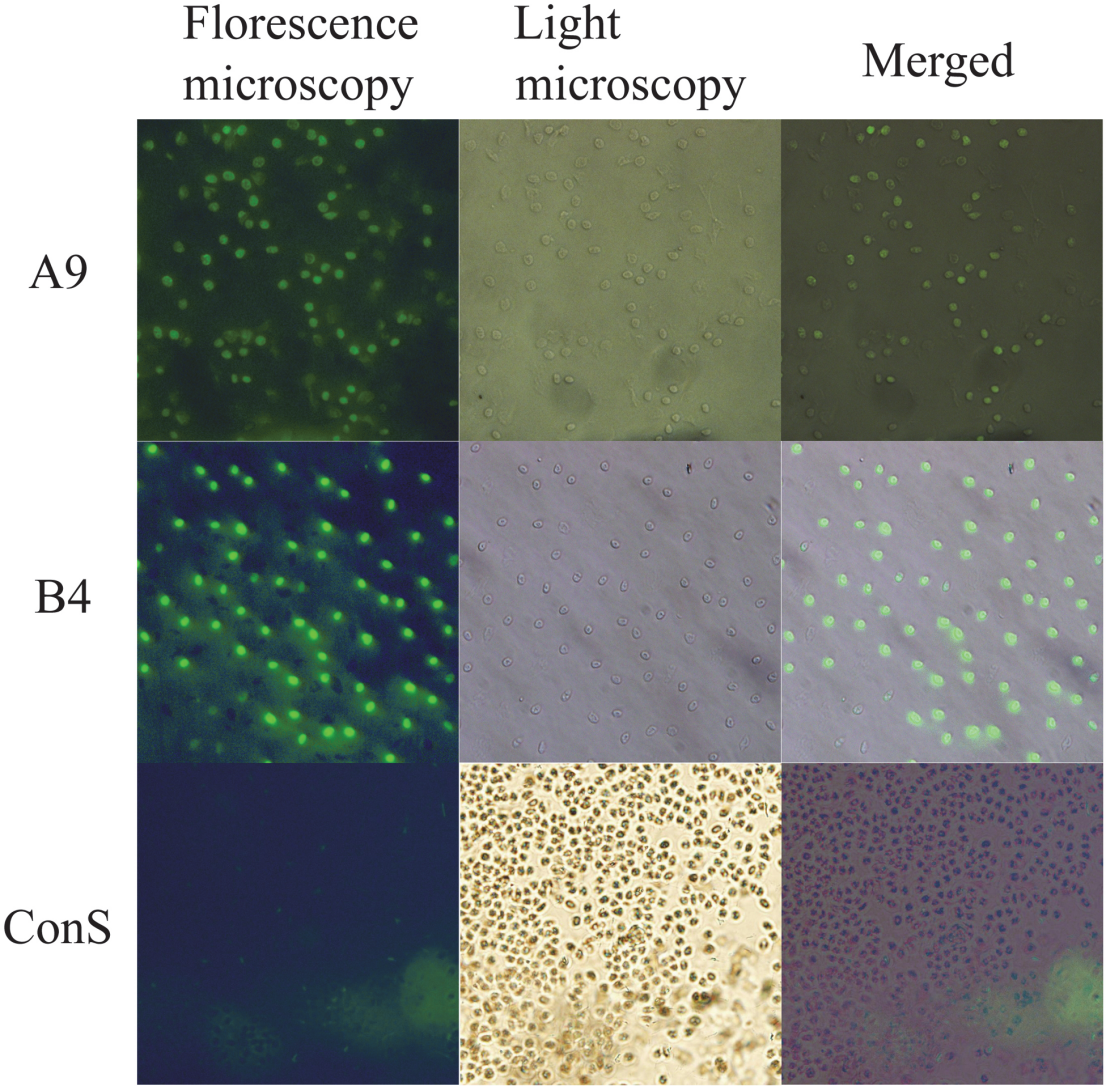
Microscopic analysis of A9 and B4 binding to the whole virus. The FAM-labeled aptamers (A9 and B4) were detected binding directly to the whole H9N2 AIVs which were absorbed to the RBC surfaces. ConS, Control Sequence labeled with FAM at 5’ terminus (5’-FAM-AGT CCG TGG TAG GGC AGG TTG GGG TGA CT-3’).

After four rounds of CE-SELEX, 32 unique sequences (S1 Table) were obtained, and of these, 5 aptamers were confirmed to have high binding affinity to purified H9-HA. As shown in Fig 2A, the CE electropherogram of the aptamer-HA complex depicted a 7.3-min migration time. Also, K_d_ values of 5 aptamers were in the nanomolar range (Table 2). However, only two aptamers (A9 and B4), were confirmed to directly bind to the whole virus and form complexes with an 8.1 min migration time (Fig 2B). K_d_ values targeting the whole virus were similar to those targeting purified HA, suggesting that A9 and B4 selected against the purified HA could also bind to whole virus. Furthermore, the high binding affinity of A9 and B4 was confirmed by ELAA, and data indicate that similar values for A9 and B4 were determined using a nonlinear least-squares regression and GraphPad Prism 5.0 (Fig 3). Finally, the ability of aptamers binding to whole virus was measured with florescent imaging, and A9 and B4 could bind to the viruses absorbed to RBC surfaces (Fig 4) as evidenced by exited fluorescence.

**Table 2:** K_d_ determination in CE.

Aptamer	K_d_ (nM)	
	Binding to HA	Binding to virus
A6	20.61 ± 7.55	-
A9	46.23 ± 5.46	42.54 ± 9.75
A21	74.27 ± 21.52	-
B4	7.38 ± 1.09	6.37 ± 0.70
B25	18.36 ± 2.10	-

### Binding specificity

**Fig 5:**
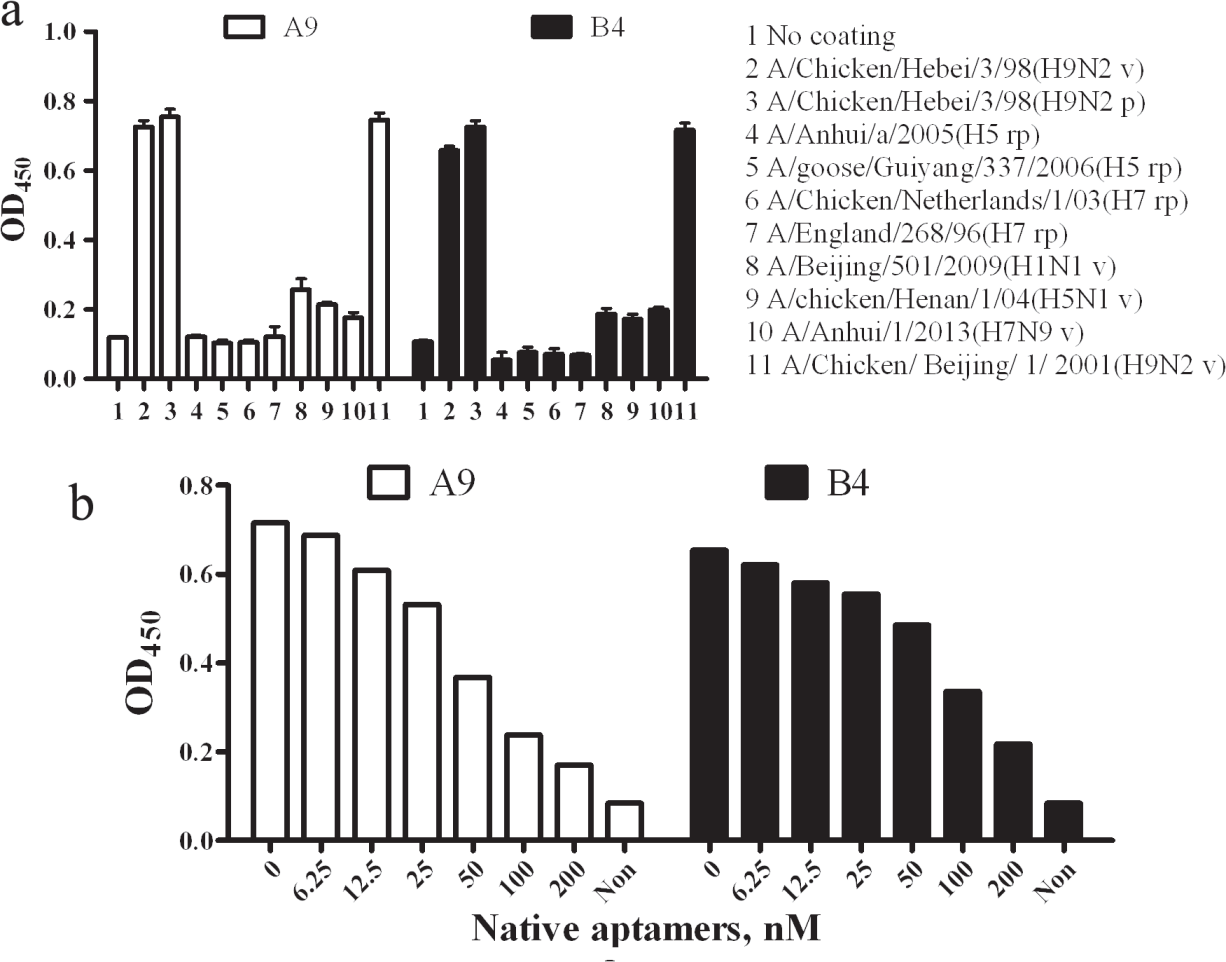
Binding specificity of A9 and B4. (a) Specificity test in ELAA using HA proteins and whole viruses of various AIV subtypes. v, whole virus; p, purified HA protein; rp, recombinant HA protein. (b) Competitive ELAA using the corresponding unlabeled aptamer. Non, none of the unlabeled aptamer and biotin labeled aptamer was used.

HA proteins or whole viruses of various AIV subtypes were used in ELAA to confirm affinity specificity of A9 and B4. Data depicted in Fig 5A suggest that aptamers incubated with the HA or subtype H9 whole AIV had higher binding affinities than aptamers incubated with other subtypes. In addition, as measured by c-ELAA, when the concentration of the unlabeled aptamers increased to 200 nM, high competitive inhibition of A9 (76.3%) and B4 (66.7%) were observed. Thus it was suggested that both A9 and B4 could specifically bind to H9 AIV.

### Predicted structures of the aptamers

**Fig 6:**
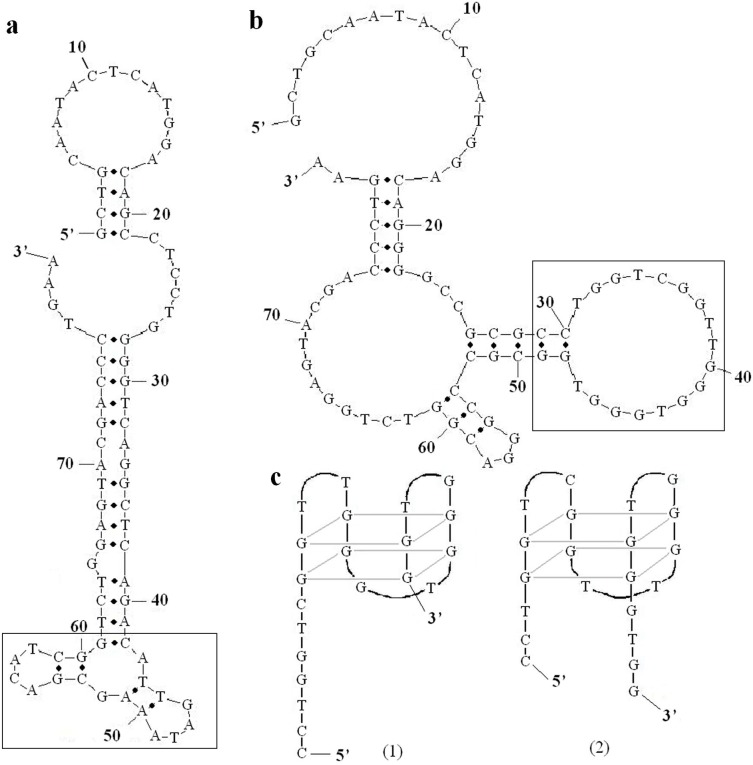
Structures of A9 and B4. (a) The predicted structures of A9. The box shows the structure of its core region. (b) The predicted structures of B4. The box shows the structure of its core region. (c) Two potential G-quadruplex structures of the sequence in the box of (b).

Potential secondary structures of A9 and B4 were analyzed with MFOLD (Fig 6), and both aptamers formed unique structures in the core region (see box). For A9, the sequence from site 42 to 61 formed a double stem-loop structure, within which the site 61 guanine nucleotide was associated with the primer region with a fixed sequence (Fig 6A). For B4, the sequence from site 29 to 49 formed a large single loop, which was suggested to have two potential G-quadruplex structures (Fig 6C). However, additional studies are needed to confirm these aptamer conformations.

### Inhibition of viral infection by aptamers

**Fig 7:**
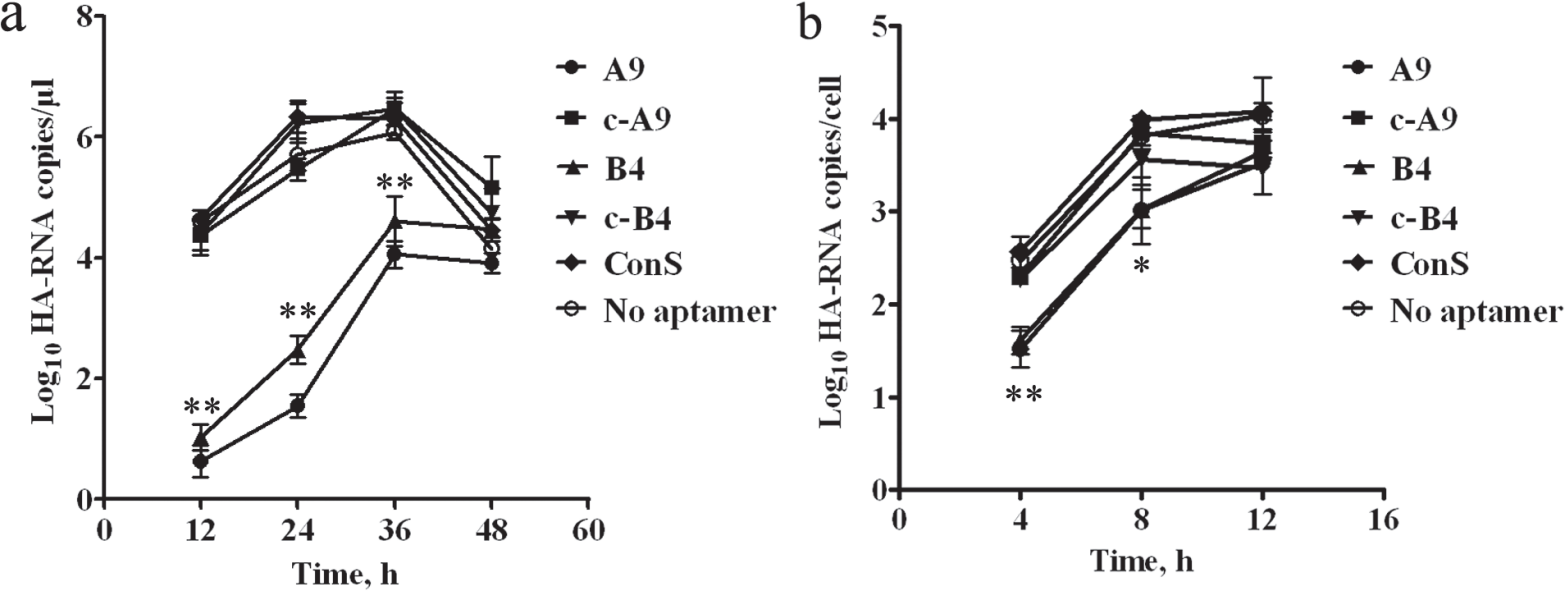
Inhibition of H9N2 virus infection in MDCK cells. (a) HA RNA quantification in supernatant. RNA was quantified with a qPCR assay at 12, 24, 36, and 48 h, respectively. The concentrations of HA-RNA in a logarithmic transformation were determined. (b) HA RNA quantification in cells. The cells harvested at 4, 8, and 12 h were quantified in a qPCR assay. The ratio of viral load to cell number, with a logarithmic transformation, was measured. Results are the mean ± SD values obtained from three independent experiments. The c-A9 represents A9 incubated with its complementary sequence, and the c-B4 represents B4 incubated with its complementary sequence. **, difference between the aptamer groups (A9 or B4) and the no aptamer groups, *P* < 0.05. *, significant difference between the aptamer groups (A9 or B4) and the no aptamer groups, *P* < 0.01. ConS, Control Sequence (5’-AGT CCG TGG TAG GGC AGG TTG GGG TGA CT-3’).

The ability of two aptamers to inhibit viral infection was verified via quantification of cellular and supernatant viral RNA. Fig 7A shows that until 36 h after infection, HA RNA copies in the supernatant of A9- and B4-treated groups were less than the no aptamer group. However, in the supernatant, RNA was not detected within 12 h post infection. Therefore, the cellular viruses were measured in the first 12 h, and were quantified using logarithmic values of HA RNA copies per cell (Fig 7B). Lower viral load was measured in A9-treated and B4-treated groups compared to the no aptamer group. Finally, the viral load recovered when the corresponding complementary A9 and B4 sequences were added.

## Discussion

Normally, one CE-SELEX round can be completed in 24 h. In our hands, aptamers were obtained in 4–5 days using 4 CE-SELEX rounds, compared to conventional SELEX which requires 2 weeks to one month to complete. Purified HA of H9N2 AIV was prepared and used to select efficient DNA aptamers. Different migration times of the aptamer-HA complex (7.3 min), H9-HA (5.3 min) and the aptamer (14.5 min) were useful for separating HA-binding aptamers from non-binding ones. Five aptamers were ultimately identified against the purified H9-HA, but only two bound to the whole virus. Possibly, binding sites of the other three aptamers were inhibited and enclosed when HA participates in viral assembly.

To quantify anti-HA aptamers activity, an HA and HA inhibition (HA/HI) test [19,22,35], along with MTT [19,21,36] and HA-mediated membrane fusion tests [23,25] were used. In this study, the quantification of the H9-HA RNA by reverse transcription qPCR was used to assay antiviral activity of A9 and B4. Result shows that the virus was measurable in the supernatant after it was detectable in cells. This may be explained by a delay in viral release from cells and the passage of time before viral concentrations could meet the detection threshold of the qPCR assay.

Finally, compared with the no aptamer group, a significantly lower ratio of viral load to cell number was observed in the groups incubated with A9 and B4, suggesting that viruses entering MDCK cells were reduced after aptamer treatment. Aptamer binding to HA likely hindered viral absorption or inhibited HA-mediated membrane fusion. However, more studies are needed to confirm the specific role of aptamers in viral invasion.

Currently, the H9N2 AIV subtype has a global presence, causing huge economic losses to the poultry industry [13], as well as representing a potential human threat via zoonotic transmission [14–15]. Anti-influenza drugs are recognized to be a primary public health strategy for controlling H9N2 AIV and presenting viral infection. Anti-HA aptamers specifically may have utility in this regard due to their small size and low incidence of adverse or immunogenic effects. Moreover, aptamers can be chemically synthesized with *in vitro* techniques, reducing bath-to-batch variation, and they can be stably stored long-term and easily transported at an ambient temperature. Meanwhile, because aptamers can be enzymatically degraded, base modification can be used to stabilize DNA and this does not alter aptamer function or therapeutic efficacy.

## Conclusions

In summary, DNA aptamers were successfully selected via CE-SELEX using purified H9-HA protein as the target. A9 and B4 were confirmed to bind to whole H9N2 virus and inhibit viral infection of MDCK cells. These results indicate that these aptamers are promising for the development of anti-AVI therapeutics for animal and human use.

## Supporting Information

S1 Table Sequences of the final 32 candidates. (DOC)
