# Supplementary material for: Two DNA Aptamers against Avian Influenza H9N2 Virus Prevent Viral Infection in Cells
Source: PLoS One. 2015 Mar 31;10(3):e0123060. doi: 10.1371/journal.pone.0123060 (PMC4380446; doi:10.1371/journal.pone.0123060)
Supplement: S1 Table — (DOC) [file pone.0123060.s001.doc]

**Supporting Information**

**Table S**1. Sequences of the final 32 candidates

| NO. | Sequence |
| --- | --- |
| A2 | **GCTGCAATACTCATGGACAG**-CCCCGTGGTGCCCCGTCCGCGACATCGCGCACATAGTGAC-**GTCTGGAGTACGACCCTGAA** |
| A3 | **GCTGCAATACTCATGGACAG**-GGACGGGGTGCTCGGAGGAGTTTTGTTCGTGGCGTTTTGC-**GTCTGGAGTACGACCCTGAA** |
| A4 | **GCTGCAATACTCATGGACAG**-CCCCGGCTTGGAAATAGAATTATGACACATTGTCACATGA-**GTCTGGAGTACGACCCTGAA** |
| A6 | **GCTGCAATACTCATGGACAG**-CGGGGGTCTCGCCCTCCCGGCGGCCTCGGCGAGCGTCGGC-**GTCTGGAGTACGACCCTGAA** |
| A7 | **GCTGCAATACTCATGGACAG**-TCCGGTTCCGGTTGGGTGTTGGCCGGGTCTGTGGGTTGTC-**GTCTGGAGTACGACCCTGAA** |
| A9 | **GCTGCAATACTCATGGACAG**-CCTCCTGGGGTCAGGCTCAGACATTGATAAAGCGACATCG-**GTCTGGAGTACGACCCTGAA** |
| A10 | **GCTGCAATACTCATGGACAG**-TGGCGCGGCTGACCAGTCTCGGTTGCCGTTGGCTTCACTG-**GTCTGGAGTACGACCCTGAA** |
| A11 | **GCTGCAATACTCATGGACAG**-CCCGTCTCGCCCTTTGTCCGGACACGCTTAGTCTGCATTC-**GTCTGGAGTACGACCCTGAA** |
| A12 | **GCTGCAATACTCATGGACAG**-GCGCGGGTTTCTGCTCTTGGGTGGGCGTCTCGTGGCGTTC-**GTCTGGAGTACGACCCTGAA** |
| A13 | **GCTGCAATACTCATGGACAG**-CGGGGCATCCTGTGGTTCCCGTTTTGGCACGACGGGTTG-**GTCTGGAGTACGACCCTGAA** |
| A14 | **GCTGCAATACTCATGGACAG**-TGCGGAGTTGGTGCGCGCGCTGGTGTCGTTTCTGCGTGGG-**GTCTGGAGTACGACCCTGAA** |
| A15 | **GCTGCAATACTCATGGACAG**-GGGCTCAGCGGCGCTTGTCTTGTCGTCTTTGTCTGTCTAC-**GTCTGGAGTACGACCCTGAA** |
| A16 | **GCTGCAATACTCATGGACAG**-GGGGGGGTTCTGGGTGTCCGTACGGGCGTTGTTTGTGTTT-**GTCTGGAGTACGACCCTGAA** |
| A18 | **GCTGCAATACTCATGGACAG**-CGACATGTTGGGTTGCCGCGTGGGTGTTCCTAACGTTCAG-**GTCTGGAGTACGACCCTGAA** |
| A19 | **GCTGCAATACTCATGGACAG**-CGCGTGGTGTCTCTGTTCGGCGTCTCGGTTAGGCGGCTCC-**GTCTGGAGTACGACCCTGAA** |
| A21 | **GCTGCAATACTCATGGACAG**-TCATGCCGGTTTCGGTCATCGCAGAAGACCCTCCGGCGAG-**GTCTGGAGTACGACCCTGAA** |
| B1 | **GCTGCAATACTCATGGACAG**-CGCGTCTGGCTCGCCCGGCGCTCTCATCCTTCGCGTCCTG-**GTCTGGAGTACGACCCTGAA** |
| B3 | **GCTGCAATACTCATGGACAG**-CGTTCGGCGGCCTGTGTTTCCTCCCGTCGTTTTGTCTGGC-**GTCTGGAGTACGACCCTGAA** |
| B4 | **GCTGCAATACTCATGGACAG**-GGGCCGCGCCTGGTCGGTTGGGTGGGTGGCGCCCGGGACG-**GTCTGGAGTACGACCCTGAA** |
| B9 | **GCTGCAATACTCATGGACAG**-TCGGCCGGCGGGTTCGGGGTGGGTCAGGCCGCTCGTCGTG-**GTCTGGAGTACGACCCTGAA** |
| B10 | **GCTGCAATACTCATGGACAG**-TCGCGGGCCCTGCCCCGTCGTGTCGCTCGGCCGTTTTTCG-**GTCTGGAGTACGACCCTGAA** |
| B11 | **GCTGCAATACTCATGGACAG**-TCCCCGGCACACGGGATTTATCAATGAACTCACGCGTGG-**GTCTGGAGTACGACCCTGAA** |
| B14 | **GCTGCAATACTCATGGACAG**-CGGGCGGGTGGAGGGGCATGCGACAGGCTGCTTGTGCCTG-**GTCTGGAGTACGACCCTGAA** |
| B17 | **GCTGCAATACTCATGGACAG**-GTGTAGCCTTAGGGCCCCTAGGCGGGCACCTCGGCTCCCT-**GTCTGGAGTACGACCCTGAA** |
| B19 | **GCTGCAATACTCATGGACAG**-CTTGTGGCCTGTGTTCCGTGGGCGTGGGAGCAGTGTCTGT-**GTCTGGAGTACGACCCTGAA** |
| B20 | **GCTGCAATACTCATGGACAG**-CCCCGCGTGTCGGTAGTTTAGGGTGTCCTCGGCTGGCCGG-**GTCTGGAGTACGACCCTGAA** |
| B21 | **GCTGCAATACTCATGGACAG**-GGGCTTCTGCTCCAGTGGCGGGTATTGTCTGCTCTCCGGG-**GTCTGGAGTACGACCCTGAA** |
| B22 | **GCTGCAATACTCATGGACAG**-GGCTGGGGGCTGAGGGGGCTCGTGAGCGGTCTAGGGGCGA-**GTCTGGAGTACGACCCTGAA** |
| B23 | **GCTGCAATACTCATGGACAG**-GGGGTGGGCCCACCTGGGATCCTGTGGCACATGGGTCTAG-**GTCTGGAGTACGACCCTGAA** |
| B25 | **GCTGCAATACTCATGGACAG**-GGTCGCCGGTTTGGCTGGCGATTCGGTTGTGTGTCAGCGT-**GTCTGGAGTACGACCCTGAA** |
| B26 | **GCTGCAATACTCATGGACAG**-GGGCCCGCCTTGTTTGATTTGCCACCCGCTTCCACGACGG-**GTCTGGAGTACGACCCTGAA** |
| B27 | **GCTGCAATACTCATGGACAG**-TGGGAGGCGCTGTGGTGGTTGTGGTGCTCCGTCGTGCGGG-**GTCTGGAGTACGACCCTGAA** |
